# Comments on Syphilinum

**Published:** 1896-05

**Authors:** H. C. Morrow

**Affiliations:** Austin, Texas


### COMMENTS ON SYPHILINUM.

H. C. Morrow, M. D., Austin, Texas.

The foregoing cases cured with Syphilinum (see The Homœopathic Physician for March, page 129) show many clinical symptoms that are not contained either in the proving published by Dr. Swan in the *Medical Advance* (Vol. XXI, p. 123), or in Hering's *Guiding Symptoms*.

With the consent of the editor and the indulgence of my readers, I have tabulated the most prominent of these, in the hope that others will add their experience, and that in time, by careful observation and comparison, our materia medica editors will know what clinical symptoms to add.

One swallow does not make a summer, nor does a clinical symptom once observed entitle it to a place in our materia medica; but when many careful, conscientious prescribers observe the same symptoms cured by the same remedy, such symptoms are then entitled to a place in the pathogenesis of the remedy under consideration. I have therefore entered the following list of symptoms under the head of

